# Increased incidence of live births in implanted day 5 versus day 6 blastocysts following single embryo transfers with PGT-A

**DOI:** 10.1038/s41598-023-40052-5

**Published:** 2023-08-05

**Authors:** Chien-Hong Chen, Chun-I Lee, Chun-Chia Huang, Hsiu-Hui Chen, Chih-Ying Chang, En-Hui Cheng, Pin-Yao Lin, Chung-I Chen, Tsung-Hsien Lee, Maw-Sheng Lee

**Affiliations:** 1 Division of Infertility, Lee Women’s Hospital, Taichung, Taiwan; 2 Department of Post-Baccalaureate Medicine, National Chung Hsing University, Taichung, Taiwan; 3 Department of Obstetrics and Gynecology, Chung Shan Medical University Hospital, Taichung, Taiwan; 4 Institute of Medicine, Chung Shan Medical University, Taichung, Taiwan

**Keywords:** Embryology, Reproductive disorders

## Abstract

Elective single-embryo transfers of euploid or low-level mosaic blastocysts were analyzed in this retrospective study to determine the correlations of live birth (LB) probability with embryonic developmental features of implanted day 5 (D5, *n* = 245) or day 6 (D6, *n* = 73) blastocysts using time-lapse (TL) monitoring. According to the logistic regression analyses (adjusted odds ratio [OR] = 0.341, 95% confidence interval [CI] = 0.169–0.685, *P* < 0.05), the LB probability was negatively associated with the D6 group. The LB rate of the D5 group was higher than the D6 group (88.2% vs. 75.3%; *P* < 0.05). Compared with the D5 blastocysts, the D6 blastocysts exhibited comparable dysmorphisms except for the multinucleation at the 4-cell stage (10.9% vs. 2.9%, *P* < 0.05). Moreover, D6 blastocysts had considerably slower developmental kinetics and poorer blastocyst morphologies. Further analysis confirmed that the LB rate was not associated with developmental kinetics or dysmorphisms but rather with blastocyst morphology (inner cell mass [ICM] grade ≤ C vs. ICM grade A, adjusted OR = 0.155, 95% CI = 0.04–0.596, *P* < 0.05; trophectoderm [TE] grade ≤ C vs. TE grade A, adjusted OR = 0.157, 95% CI = 0.032–0.760, *P* < 0.05). In conclusion, D6 implanted blastocysts have a considerably lower LB rate than D5 implanted blastocysts. As determined by TL monitoring, the diminished blastocyst morphology can be one of the primary reasons underlying the decreased likelihood of LB.

## Introduction

Although randomized controlled trials have revealed no difference in pregnancy outcomes between frozen and fresh embryos^1,2^, advances in embryonic cryopreservation technology, specifically the development of vitrification techniques, have not only greatly increased the safety and usefulness of embryo freezing but also helped women seeking to preserve their fertility by promoting the widespread use of blastocyst frozen embryo transfer (FET)^3^. Research has also indicated that endometrial receptivity is adversely affected by ovarian stimulation during in vitro fertilization (IVF) procedures, which have a negative effect on endometrial normality and influence endometrial development^4,5^. These findings indicate that FET may be advantageous for endometrial–embryonic synchronization during IVF cycles.

High-resolution next-generation sequencing (hr-NGS) has been increasingly used worldwide in preimplantation genetic testing for aneuploidy (PGT-A) because of its high precision, effectiveness, and throughput^6,7^. When combined with the FET strategy, embryo selection through PGT-A can substantially improve the implantation outcomes of single-embryo transfer (SET) cycles^8^. Cytogenetic studies have indicated that approximately 50% of miscarriage samples contain chromosomal abnormalities, which are regarded as the most critical cause of spontaneous miscarriages^9,10^. Specifically, advanced maternal age has been associated with a sharp increase in miscarriages^11^. In IVF cycles, the application of PGT-A has been reported to effectively mitigate the risk of miscarriage^12–14^. However, miscarriages are still maintained at a low level after the implantation of euploid embryos, and their underlying causes remain unclear^15^.

Recent studies on embryo–endometrium interactions have indicated that women with a risk of miscarriage may be less selective than their counterparts in terms of embryo implantation. During peri-implantation, embryonic signals reach the decidualized endometrium and stimulate endometrial cell migration, and the biosensor of these endometrial cells selects a qualified blastocyst for implantation^16–19^. Therefore, selecting the most viable embryo before embryo transfer (ET) is essential to mitigate the miscarriage risk of successfully implanted embryos in PGT-A cycles.

Accordingly, selecting qualified frozen blastocysts is critical for optimizing the pregnancy outcomes of FET. Numerous studies have indicated that fast-growing blastocysts are associated with better clinical outcomes than those of slow-growing blastocysts. They have also indicated that the differences in the embryonic factors between fast-growing and slow-growing blastocysts are a topic that warrants further study^20–23^. Time-lapse (TL) monitoring has been used in IVF to provide a detailed and dynamic evaluation of the kinetics, dysmorphisms, and morphology of fertilized embryos, all of which have been proposed as being able to predict embryo growth, ploidy status, and pregnancy success^24–27^. The primary objective of the present study was to evaluate the TL data of individual implanted embryos in PGT-A cycles to determine whether developmental kinetics, cleavage anomalies, and blastocyst morphologies are related to the likelihood of fetal loss (FL) or live birth (LB). These TL features can be used in noninvasive analyses to improve the selection of competent embryos that may have high LB potential after implantation. This may be particularly useful for IVF patients who have a risk of miscarriage.

## Materials and methods

### Study design

This retrospective cohort study was performed in accordance with relevant guidelines and regulations. The Institutional Review Board of Chung Shan Medical University (approval number CS1-21156) provided a waiver of written informed consent for this study. Data on pregnant women undergoing ET of frozen–thawed blastocysts after PGT-A cycles were gathered from Lee Women’s Hospital from January 2018 to December 2021. Patients with an endometrial thickness of 7 mm or less, severe uterine abnormalities, transfers of more than one embryo, and a transferred embryo with a mosaic level of 50% or higher were excluded. A total of 318 FET cycles, in which intrauterine pregnancies were confirmed by visualizing at least one gestational sac, from 304 patients were included. The baseline characteristics and FET cycle parameters were collected, including the number of previous IVF cycles, age, anti-Müllerian hormone (AMH) level, body mass index (BMI), serum estradiol (E2), and progesterone (P4) levels on the day of ET, oocyte sources, sperm quality, endometrial preparation protocols, embryo ploidy status, and blastocyst vitrification day.

### Laboratory procedures

All laboratory procedures were conducted in accordance with the standard protocols described in our previous reports^25,26^. Briefly, a gonadotrophin-releasing hormone (GnRH) agonist long protocol (Lupron; Takeda Chemical Industries, Osaka, Japan) or a GnRH antagonist protocol (Cetrotide; Merck Serono, Geneva, Switzerland) was used for controlled ovarian stimulation. Follicle development was stimulated by the administration of exogenous gonadotropin (GONAL-f, Merck Serono; Menopur; Ferring Pharmaceuticals, São Paulo, Brazil) until the size of the leading follicle reached or exceeded 18 mm. Human chorionic gonadotropin (hCG, 250 μg, Ovidrel; Merck Serono, Modugno, Italy) was then used to stimulate oocyte maturation, and ultrasound-guided ovum retrieval was conducted 36 h after the administration of hCG. The derived oocytes inseminated by the method of intracytoplasmic sperm injection or conventional insemination were cultured in a TL culture system (EmbryoScope + ; Vitrolife, Kungsbacka, Sweden) with sequential media (SAGE Biopharma, Bedminster, NJ, USA) in an environment containing 5% O_2_, 5% CO_2_, and 90% N_2_ at 37 °C. TL assessments of individual embryos for morphokinetics, cleavage dysmorphisms, and morphology were subsequently performed at 118 h post insemination (hpi) by using all of the recorded TL images in accordance with the process outlined in our previous report^26^. Briefly, the blastocyst expansion levels were annotated to highlight the specific developmental features, including a blastocoel cavity beginning to form (level 1), to expand (level 2), and to herniate (level 3). Additionally, the evaluations of ICM and TE quality were conducted following the manufacturer's guidelines, which were applied to generalize the Gardner blastocyst grading system to time-lapse imaging. Detailed information is provided in Supplementary Table 1.

Qualified blastocysts on day 5 (D5) or day 6 (D6) expanded blastocysts (embryo diameter ≥ 150 µm) with an inner cell mass (ICM) grade of at least B or a trophectoderm (TE) grade of at least B were selected for embryo biopsy. Micromanipulation with inverted microscopy and a laser system was applied to carefully separate 5 to 8 trophectoderm (TE) cells from a blastocyst. The separated TE cells were rinsed with phosphate-buffered saline thoroughly and then cautiously placed on the bottom of an RNAse–DNAse-free polymerase chain reaction tube for the following tests. The remaining blastocysts were incubated in vitro for at least 3 h prior to cryopreservation. An hr-NGS platform (Illumina, San Diego, CA, USA) was used to determine the mosaic levels of biopsied blastocysts as per the manufacturer’s instructions.

### Embryo cyropreservation and FET

Vitrification and warming of the biopsied blastocysts were accomplished using the Cryotech method (Cryotech, Tokyo, Japan). Women undergoing FETs of a single blastocyst selected on the basis of hr-NGS results and blastocyst morphology were subjected to a natural (NC), modified natural (mNC), or artificial (AC) cycle of endometrial preparation. The ovulation of the dominant follicle in a NC was monitored by transvaginal ultrasound detection. A mNC was defined by triggering the ovulation of the leading follicle (≥ 18 mm) using hCG injection (250 μg, Ovidrel; Merck Serono, Modugno, Italy). Luteal-phase support (LPS) for NC and mNC was offered from the first day after ovulation to the day of the pregnancy test, including oral dydrogesterone 10 mg three times a day (Duphaston, Abbott Biologicals B.V., the Netherlands), vaginal micronized progesterone 90 mg two times a day (Crinone 8%, Merck Serono, Darmstadt, Germany), and oral estradiol valerate 6 mg daily (Estrade, Synmosa, Taipei, Taiwan). In the AC, the patients were administered with an estradiol valerate supplementation regimen of endometrial preparation as follows: (1) 4 mg daily on days 3–4 of their natural menstrual cycle; (2) 8 mg daily on days 5–7 of their natural menstrual cycle; (3) 12 mg daily on days 8–12 of their natural menstrual cycle. From day 13 of the menstrual cycle to the day of the pregnancy test, LPS for an AC was offered for patients with sufficient endometrial thickness, including oral dydrogesterone 10 mg three times a day, vaginal micronized progesterone 90 mg three times a day, and oral estradiol valerate 6 mg daily. The embryo transfer was performed on day 5 after ovulation in the NC and mNC or after progesterone administration in the AC for the patients with endometrial thickness of at least 8 mm. If the endometrial thickness was less than 8 mm, the transfer was canceled and shifted to the next cycle. The LPS with oral dydrogesterone and vaginal micronized progesterone continued up to 10 weeks of gestation for pregnant patients.

On the ET day, the endometrial thickness and serum E2 and P4 levels were measured for each patient, and then the clinical outcomes of pregnant patients with a visualized intrauterine gestational sac were evaluated. A LB was defined as a baby born alive at 24 weeks of gestation or more. A FL was defined as a pregnancy characterized by the occurrence of a blighted ovum, absence of a fetal heartbeat, intrauterine fetal death or growth restriction, or stillbirth (fetal death at 20 weeks of gestation or more).

### Statistical analysis

All statistical analyses were performed using IBM SPSS Statistics version 20.0 (IBM, Armonk, NY, USA) or GraphPad Prism version 6.0 h (GraphPad Software, San Diego, CA, USA). Proportions were used to summarize categorical variables, and means with standard deviations were used to summarize continuous variables. Generalized estimating equation (GEE) analysis with logistic regression settings was used to assess the correlations between the LB probability and independent variables in unadjusted (univariate) and adjusted (multivariate) models. The confounders were determined by the backward elimination procedure until the remaining variables in the multivariate regression model had a *P* value < 0.2. The differences between groups were assessed using the Kolmogorov–Smirnov test, Fisher’s exact test, or chi-square test, as applicable. Statistical significance was set at *P* < 0.05 in all analyses.

### Ethics approval and consent to participate

This retrospective cohort study was reviewed and approved by the Institutional Review Board of Chung Sun Medical University, Taichung, Taiwan (Approval Number CS1-21156).

## Results

### Potential factors influencing the LB probability of implanted blastocysts

**Figure 1:**
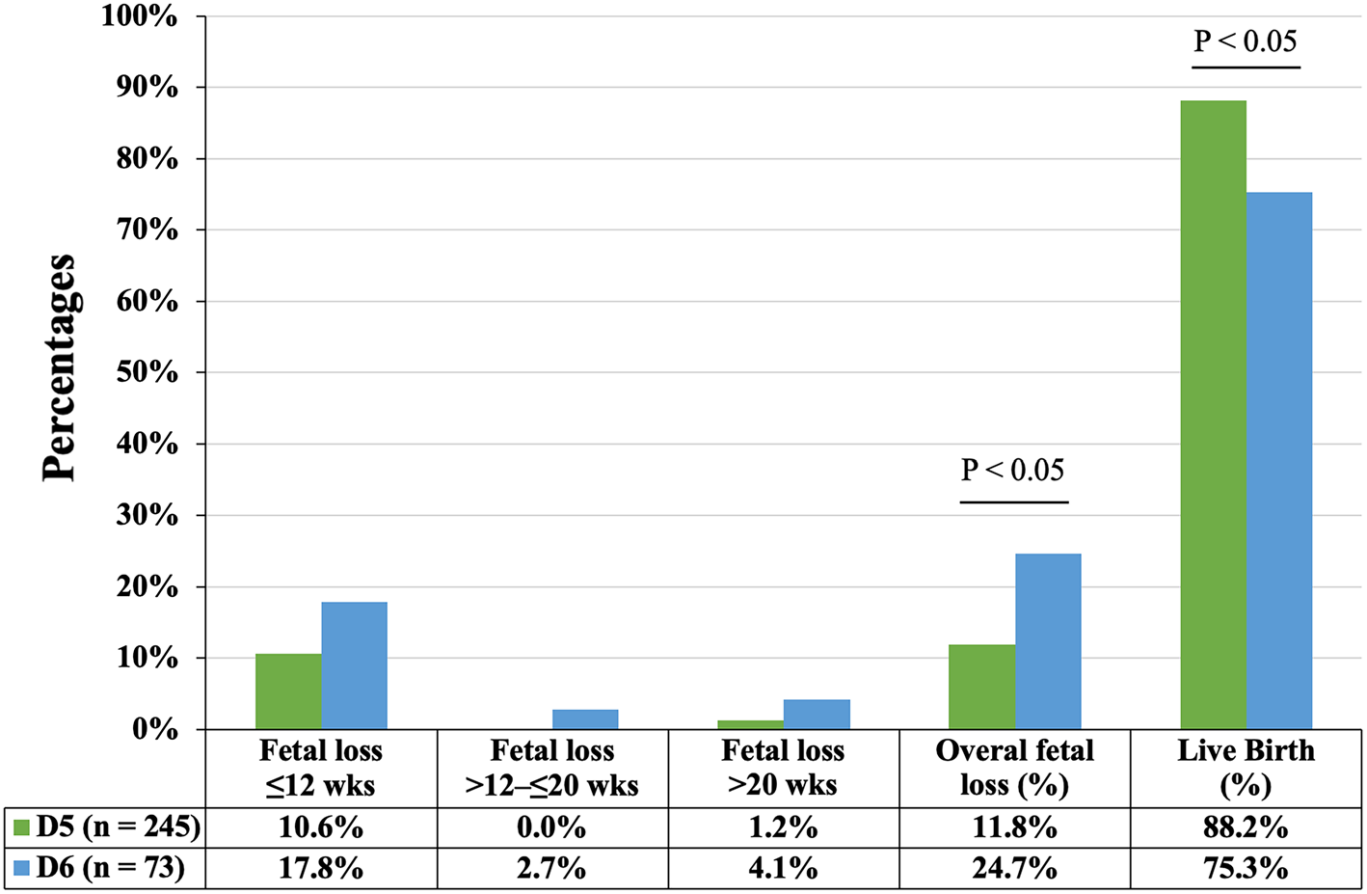
The postimplantation outcomes of day 5 vs. day 6 blastocysts. Following embryo transfer and implantation, Fisher’s exact test was used to compare the fetal loss and live birth rates between day 5 and day 6 groups. Abbreviations “wks”, “D5”, and “D6” denoted the weeks of gestation, day 5, and day 6, respectively.

Women (n = 304) who underwent FETs with single euploid or low-level mosaic blastocysts and who exhibited a positive sign of pregnancy through gestational sac visualization 5 weeks after their last menstrual period were included in this study. Of the cohort of 318 SETs, 245 women successfully gave birth. Univariate logistic analysis with the GEE model revealed that none of the patient characteristics were correlated with the probability of LB, i.e., the number of previous IVF cycles, age, AMH level, BMI, E2, and P4 levels on the day of ET, oocyte sources, sperm quality, endometrial preparation protocols, and embryo ploidy status, with the exception of the blastocyst vitrification day. Compared with ETs with a D5 blastocyst (i.e., the D5 group), ETs with a D6 blastocyst (i.e., the D6 group) were negatively correlated with the probability of LB (odds ratio [OR] = 0.41, 95% confidence interval [CI] = 0.207–0.813; *P* = 0.011; Table 1). When taking consideration of the confounders (i.e., AMH, E2 levels, oocyte sources, and ploidy status) determined by the backward elimination procedure, ETs with a D6 blastocyst were still negatively correlated with the probability of LB (adjusted OR = 0.341, 95% CI = 0.169–0.685; *P* = 0.003; Table 1). In addition, the D5 group had lower rates of FL at ≤ 12 weeks (10.6% versus [vs.] 17.8%), > 20 to ≤ 20 weeks (0% vs. 2.7%), and > 20 weeks (1.2% vs. 4.1%) than those of the D6 group, which resulted in a significant increase in the LB rate in the D5 group (88.2% vs. 75.3%; *P* < 0.05; Fig. 1).

**Table 1:** The correlations between live birth probabilities and cycle variables were determined using logistic regression analysis in the generalized estimating equation model.

Variables	Univariate				Multivariate			
	Odds ratio	95% Confidence interval		*P* value	Adjusted odds ratio	95% Confidence interval		*P* value
		Lower	Upper			Lower	Upper	
Previous IVF cycle numbers	1.020	0.940	1.107	0.629	–	–	–	–
Female age (recipient, years)	1.016	0.963	1.071	0.562	–	–	–	–
Female age (oocyte, years)	0.978	0.928	1.031	0.409	–	–	–	–
AMH (ng/mL)	0.930	0.863	1.002	0.057	0.903	0.836	0.977	0.011
BMI (kg/m^2^)	1.011	0.924	1.107	0.811	–	–	–	–
E2 (ET day, pg/mL)	1.000	0.999	1.000	0.233	1.000	0.999	1.000	0.104
P4 (ET day, ng/mL)	1.001	0.993	1.009	0.834	–	–	–	–
Oocyte sources, OD vs. AT	1.909	0.597	6.101	0.275	2.297	0.568	9.289	0.243
Oocyte sources, EGB vs. AT	2.005	0.450	8.934	0.362	3.288	0.672	16.081	0.142
Sperm quality, normal vs. abnormal	1.358	0.703	2.623	0.362	–	–	–	–
Endometrial preparation, NC and mNC vs. AC	0.713	0.376	1.354	0.302	–	–	–	–
Ploidy status, mosaic vs. euploid	3.077	0.933	10.153	0.065	3.603	0.985	13.179	0.053
Vitrification day, D6 vs. D5	0.410	0.207	0.813	0.011	0.341	0.169	0.685	0.003

The abbreviations “IVF”, “AMH”, “BMI”, “E2”, “P4”, “OD”, “AT”, “EGB”, “AC”, “NC”, “mNC”, “D6”, and “D5” denoted in vitro fertilization, anti-Mullerian hormone, body mass index, serum estradiol, serum progesterone, oocyte donation, autologous, egg bank, artificial cycle, natural cycle, modified natural cycle, day 6, and day 5, respectively.

### Baseline characteristics of transfer cycles with D5 and D6 implanted blastocysts

Table 2 presented the differences in the patient characteristics between the D5 and D6 groups. The Kolmogorov–Smirnov test revealed that the female age (oocytes and recipients), AMH level, BMI, E2, and P4 levels on the FET day, number of previous IVF cycles, and infertility periods did not differ between the two groups. The chi-square test or Fixher’s exact test revealed no differences in infertility status, oocyte sources, sperm quality, endometrial preparation, or ploidy status (Table 2).

**Table 2:** Comparisons of cycle characteristics between the implanted day 5 and day 6 blastocyst groups.

Groups	Day 5 (n = 245)	Day 6 (n = 73)	*P* value
Female Age (oocyte, mean ± SD)	33.5 ± 5.6	34.9 ± 5.7	NS
Female Age (recipient, mean ± SD)	36.4 ± 5.3	37.3 ± 5.2	NS
AMH (ng/mL, mean ± SD)	4.8 ± 3.8	4.2 ± 3.3	NS
BMI (Kg/m^2^, mean ± SD)	22.2 ± 3.4	21.8 ± 2.9	NS
E2 (pg/mL, mean ± SD)	436.8 ± 558.8	388.4 ± 330.9	NS
P4 (ng/mL, mean ± SD)	39.8 ± 35.0	34.6 ± 22.6	NS
Previous IVF cycles (mean ± SD)	3.7 ± 3.3	4.6 ± 4.1	NS
Infertility periods (years, mean ± SD)	2.9 ± 3.1	2.9 ± 2.5	NS
Infertility status (%)			NS
Tubal factors	6 (2.4)	3 (4.1)	
Ovarian factors	51 (20.8)	10 (13.7)	
Male factors	6 (2.4)	3 (4.1)	
Multiple factors	172 (70.2)	55 (75.3)	
Unknown factors	10 (4.1)	2 (2.7)	
Oocyte sources (%)			NS
AT	200 (81.6)	62 (84.9)	
OD	29 (11.8)	4 (5.5)	
EGB	16 (6.5)	7 (9.6)	
Sperm quality (%)			NS
Normal	167 (68.2)	48 (65.8)	
Abnormal	78 (31.8)	25 (34.2)	
Endometrial preparation (%)			NS
NC and mNC	75 (30.6)	20 (27.4)	
AC	170 (69.4)	53 (72.6)	
Ploidy status (%)			NS
Euploid	210 (85.7)	58 (79.5)	
Mosaic	35 (14.3)	15 (20.5)	

The abbreviations “SD”, ”NS” “AMH”, “BMI”, “E2″, “P4″, “OD”, “AT”, “EGB”, “AC”, “NC”, and “mNC” denoted standard deviation, non-significance, anti-Mullerian hormone, body mass index, serum estradiol, serum progesterone, oocyte donation, autologous, egg bank, artificial cycle, natural cycle, and modified natural cycle, respectively. The differences between groups were evaluated using Kolmogorov–Smirnov test, chi-square test, or Fisher's exact test, as appropriate.

### Embryological characteristics of D5 and D6 implanted blastocysts

TL monitoring was used to evaluate the differences in the embryological characteristics between the D5 and D6 groups. Fisher’s exact test or chi-square test was used to determine the differences in embryonic dysmorphism between the groups. The results indicated that the incidence of embryonic dysmorphism in the implanted embryos was typically less than 10%. However, TL monitoring revealed an increased frequency of uneven cleavage at the four-cell stage (13.7% to 15.9%), multinucleation at the two-cell stage (21.6% to 24.7%), noncentral juxtaposition (74% to 76.3%), uneven pronuclear size (39.7% to 42%), and vacuolization (9% to 10.9%). A comparison of the D5 and D6 blastocysts revealed that the proportions of embryonic dysmorphisms (i.e., uneven cleavage at the two-cell stage, uneven cleavage at the four-cell stage, multinucleation at the two-cell stage, noncentral juxtaposition of pronuclei, no pronuclear contact, uneven pronuclear size, unsynchronized pronuclear fading, twist-and-crumble division, incomplete chaotic division, direct unequal cleavage, reverse cleavage, delayed cleavage, vacuolization, and premature compaction) were not significantly different between the groups (Table 3). However, the rate of multinucleation at the 4-cell stage (10.9% vs. 2.9%) was significantly higher in the D6 group than that in the D5 group.

**Table 3:** The differences of embryonic dysmorphisms between implanted day 5 and day 6 blastocysts.

Groups	Day 5 (n = 245)	Day 6 (n = 73)	*P* value
Uneven cleavage (%)			
2-cell	16 (6.5)	3 (4.1)	NS
4-cell	39 (15.9)	10 (13.7)	NS
Multinucleation (%)			
2-cell	53 (21.6)	18 (24.7)	NS
4-cell	7 (2.9)	8 (10.9)	< 0.01
Non-central juxtaposition (%)	187 (76.3)	54 (74.0)	NS
No pronuclear contact (%)	7 (2.9)	3 (4.1)	NS
Uneven PN size (%)	103 (42.0)	29 (39.7)	NS
Unsynchronized PN fading (%)	1 (0.4)	0 (0)	NS
Twist-and-crumble division (%)	14 (5.7)	3 (4.1)	NS
Incomplete chaotic division (%)	6 (2.5)	3 (4.1)	NS
Direct unequal cleavage (%)	1 (0.4)	2 (2.7)	NS
Reverse cleavage (%)	8 (3.3)	1 (1.4)	NS
Delayed cleavage (%)	5 (2.0)	1 (1.4)	NS
Vacuolization (%)	22 (9.0)	8 (10.9)	NS
Premature compaction (%)	4 (1.6)	2 (2.7)	NS

The differences between groups were evaluated using chi-square test or Fisher's exact test, as appropriate. The abbreviation ”NS” denoted non-significance. The dysmorphisms were defined in the Supplementary Table 1.

After pronuclear fading, the embryonic kinetics of the D5 group were faster than those of the D6 group. Specifically, significant differences were observed between the groups in t3 (13.9 ± 1.3 h vs. 15.5 ± 1.5 h), t4 (14.8 ± 2.3 h vs. 16.5 ± 1.9 h), t5 (27.6 ± 2.8 h vs. 31.7 ± 4.7 h), t8 (32.7 ± 6.5 h vs. 37.6 ± 6.9 h), tM (62.8 ± 6.9 h vs. 70.9 ± 6.9 h), tSB (71.6 ± 4.7 h vs. 79.6 ± 6.5 h), and tB (80.2 ± 5.0 h vs. 90.3 ± 7.2 h). In addition, blastocyst formation took longer in the D6 group (tSB–tB, 10.7 ± 4.2 h) than in the D5 group (tSB–tB, 8.7 ± 2.9 h; *P* < 0.05). When the KIDScore™ D5 algorithm was used, these differences in embryonic kinetics resulted in considerably lower scores in the D6 group (2.9 ± 1.6) than in the D5 group (4.8 ± 1.3; Table 4).

**Table 4:** The differences of embryonic morphokinetics and KIDScore™ D5 scores between implanted day 5 and day 6 blastocysts.

Groups	Day 5 (n = 245)	Day 6 (n = 73)	*P* value
t2	2.7 ± 0.5	2.8 ± 0.7	NS
t3	13.9 ± 1.3	15.5 ± 1.5	< 0.001
t4	14.8 ± 2.3	16.5 ± 1.9	< 0.001
t5	27.6 ± 2.8	31.7 ± 4.7	< 0.001
t8	32.7 ± 6.5	37.6 ± 6.9	< 0.001
tM	62.8 ± 6.9	70.9 ± 6.9	< 0.001
tSB	71.6 ± 4.7	79.6 ± 6.5	< 0.001
tB	80.2 ± 5.0	90.3 ± 7.2	< 0.001
tM–tB	17.5 ± 6.0	19.4 ± 5.9	NS
tSB–tB	8.7 ± 2.9	10.7 ± 4.2	< 0.001
KIDScore™ D5 scores	4.8 ± 1.3	2.9 ± 1.6	< 0.001

The differences between groups were evaluated using Kolmogorov–Smirnov test. The abbreviation ”NS” denoted non-significance. The morphokinetic parameters were defined in Supplementary Table 1.

The components of the blastocyst morphology were redefined through a detailed evaluation completed using TL monitoring at a specific time window (118 hpi). Substantial differences were noted between the D5 and D6 groups in terms of expansion level ≥ 2 (99.2% vs. 78.1%), ICM level ≥ B (98.4% vs. 74.0%), and TE level ≥ B (92.7% vs. 46.6%; Table 5).

**Table 5:** The differences of blastocyst morphology between implanted day 5 and day 6 blastocysts using time-lapse monitoring at 118 h post insemination.

Groups	Day 5 (n = 245)	Day 6 (n = 73)	*P* value
Expansion levels			< 0.01
≤ 1 (%)	2 (0.8)	16 (21.9)	
2 (%)	189 (77.2)	56 (76.7)	
3 (%)	54 (22.0)	1 (1.4)	
ICM levels			< 0.01
A and B (%)	241 (98.4)	54 (74.0)	
≤ C (%)	4 (1.6)	19 (26.0)	
TE levels			< 0.01
A and B (%)	227 (92.7)	34 (46.6)	
≤ C (%)	18 (7.3)	39 (53.4)	

The differences between groups were evaluated using chi-square test or Fisher's exact test, as appropriate. The components of blastocyst morphology were defined in Supplementary Table 1.

### Embryological factors influencing the LB probability of implanted blastocysts

**Figure 2:**
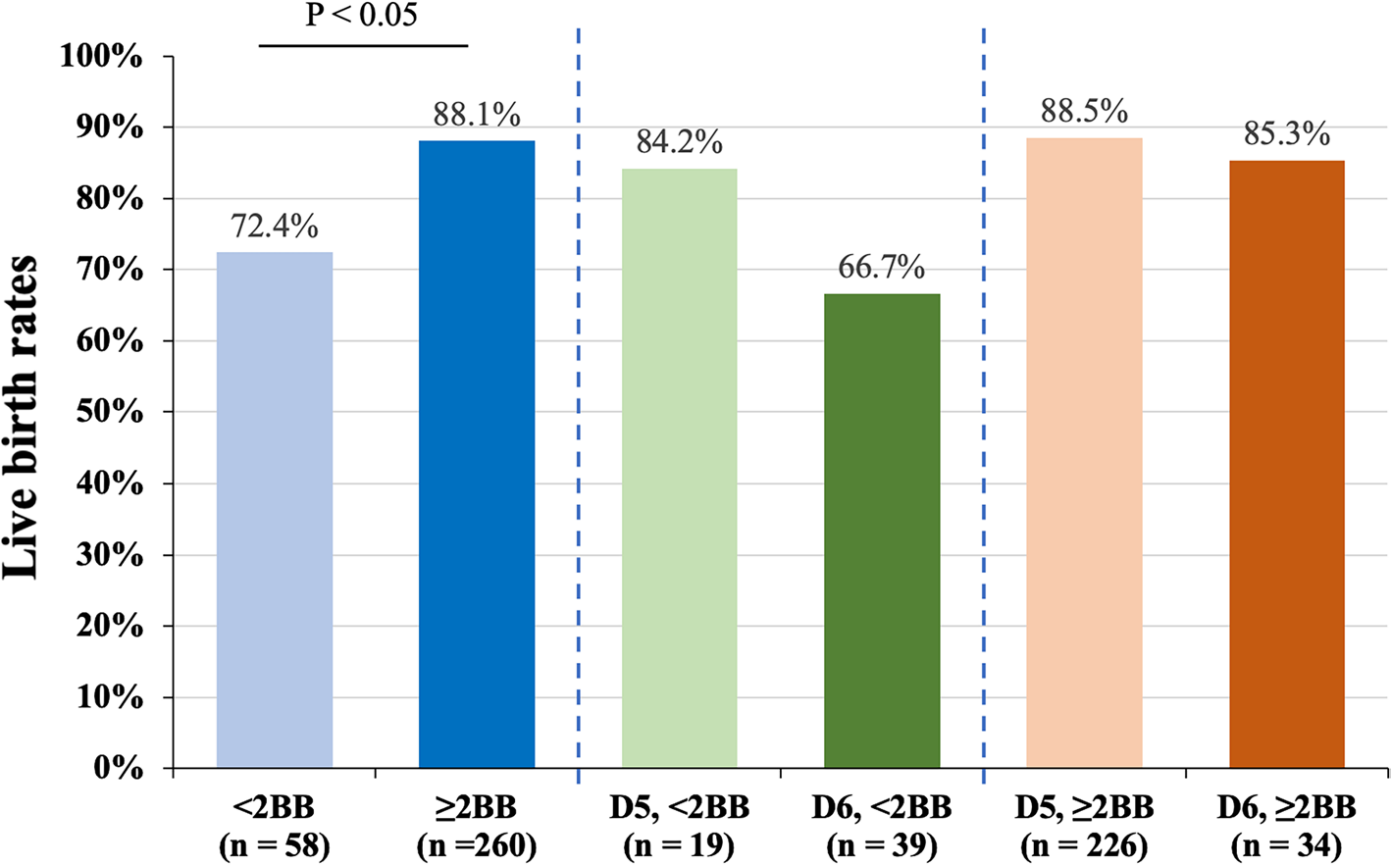
The live birth rates of implanted blastocysts stratified by embryo morphology. Following embryo transfer and implantation, Fisher exact test was used to compare the live birth rates of implanted blastocysts with morphology < 2BB or ≥ 2BB. The abbreviation “2BB” denoted blastocysts with the level 2 of expansion, the grade B of inner cell mass, and the grade B of trophectoderm. The abbreviations “D5” and “D6” denoted day 5, and day 6, respectively.

Logistic analysis with the GEE model was used to determine the correlation between the probability of LB and embryological factors, which varied between the D5 and D6 groups. The results revealed that multinucleation at the 4-cell stage, embryonic developmental kinetics, KIDScore™ D5 scores, and expansion levels were not significantly associated with the probability of LB (Tables 6 and 7). However, compared with ICM level A, ICM levels ≤ C were negatively correlated with LB in univariate (OR = 0.179, 95% CI = 0.049–0.656, *P* = 0.009) and multivariate (adjusted OR = 0.155, 95% CI = 0.04–0.596, *P* = 0.007) logistic regression models. Compared with TE level A, TE levels ≤ C were negatively correlated with LB in univariate (OR = 0.226, 95% CI = 0.061–0.844, *P* = 0.027) and multivariate (adjusted OR = 0.157, 95% CI = 0.032–0.760, *P* = 0.021) logistic regression models (Table 7). A blastocyst morphology ≥ 2BB was defined as a favorable morphology. As indicated in Fig. 2, the LB rate was considerably higher in blastocysts with a favorable morphology (≥ 2BB, 88.1%) than in those with a poor morphology (< 2BB, 72.4%). However, a comparison of D5 and D6 blastocysts with the same embryonic morphology indicated that the LB rate did not differ between the poor-morphology (84.2% vs. 66.7%) and favorable-morphology (88.5% vs. 85.3%) blastocyst groups (*P* > 0.05).

**Table 6:** The correlations of live birth probabilities with developmental dysmorphisms and kinetics were determined using univariate logistic regression analysis in the generalized estimating equation model.

Variables	Live birth probabilities			
	Odds ratio	95% Confidence interval		*P* value
		Lower	Upper	
Multinucleation at the 4-cell stage	1.134	0.246	5.217	0.872
t3	1.007	0.764	1.327	0.962
t4	1.042	0.891	1.219	0.605
t5	0.949	0.874	1.029	0.206
t8	1.008	0.962	1.056	0.749
tM	0.979	0.938	1.020	0.311
tSB	0.964	0.914	1.016	0.172
tB	0.979	0.936	1.025	0.365
tSB-tB	1.038	0.937	1.150	0.471
KIDScore™ D5 scores	1.196	0.994	1.439	0.058

The definitions of developmental kinetics were described in Supplementary Table 1.

**Table 7:** The correlations between live birth probabilities and the components of blastocyst morphology were determined using logistic regression analysis in the generalized estimating equation model.

Variables	Univariate				Multivariate			
	Odds ratio	95% Confidence interval		*P* value	Adjusted odds ratio	95% Confidence interval		*P* value
		Lower	Upper			Lower	Upper	
Expansion level ≤ 1	0.391	0.119	1.286	0.122	0.390	0.120	1.263	0.116
Expansion level 2	1.302	0.606	2.801	0.499	1.338	0.601	2.981	0.476
Expansion level 3	1	–	–	–	1	–	–	–
ICM level ≤ C	0.179	0.049	0.656	0.009	0.155	0.040	0.596	0.007
ICM level B	0.308	0.118	0.799	0.016	0.262	0.089	0.766	0.014
ICM level A	1	–	–	–	1	–	–	–
TE level ≤ C	0.226	0.061	0.844	0.027	0.157	0.032	0.760	0.021
TE level B	0.618	0.183	2.080	0.437	0.475	0.105	2.159	0.336
TE level A	1	–	–	–	1	–	–	–

Adjusted odds ratio: the odds ratio was adjusted by AMH, serum estradiol levels, oocyte sources, and ploidy status. The components of blastocyst morphology were defined in the Supplementary Table 1.

## Discussion

In our previous study^28^, in which a hr-NGS platform was used for PGT-A, we reported that FET groups of euploid and low-level mosaic blastocysts have comparable abortion rates. This means that the transfer of low-level mosaic embryos results in the birth of healthy, viable children. In the present study, we investigated the factors that likely influence the postimplantation development of euploid or low-level mosaic blastocysts in FET cycles. Logistic regression analysis with and without controlling for confounders revealed a correlation between the LB probability of implanted frozen–thawed embryos and blastocysts developing on D5 or D6 (Table 1). According to array comparative genomic hybridization and single-nucleotide polymorphism data, the LB rate per FET cycle is considerably higher in D5 euploid blastocysts than in D6 euploid blastocysts^29–31^. Even when confounders are adjusted for, the associations between the blastocyst development rate and clinical outcomes remain notable^29^. However, given the similar FL rates, the differences in the LB rates of successfully implanted embryos are nonsignificant between D5 and D6 euploid transfers^29,30,32^. In our clinical setting, we discovered that the D6 blastocysts were associated with an increased risk of FL in pregnant patients who received euploid or low-level mosaic blastocysts, which resulted in a considerably lower LB rate of D6 blastocyst transfers compared with D5 blastocyst transfers (Fig. 1). Generally, delayed blastocyst development is associated with an increased risk of mosaicism^26^, indicating that the incidence of aberrant ploidy in D6 blastocysts may increase and thereby lead to an increased risk of FL in non-PGT-A cycles^33–35^. However, according to our findings, the difference in ploidy status between the D5 and D6 blastocysts may not be the only intrinsic factor influencing the postimplantation development of embryos. Therefore, further understanding the differences between blastocysts developing on D5 and D6 through TL monitoring is essential. Embryonic factors may also serve as biomarkers for embryo selection that can be used to mitigate the risk of FL after implantation.

Several in vitro coculture studies have supported the concept of embryo–endometrium interactions in the selection of potent embryos for implantation. According to these studies, the production of implantation regulators of decidualized endometrial cells, such as the cytokines of interleukin (IL)-1β, heparin-binding epidermal growth factor-like growth factor, IL-6, and IL-10, is substantially inhibited in developmentally impaired human embryos^16^. In cocultures of embryos with a poor morphology, the migratory response of decidualized endometrial cells derived from normally fertile women is diminished^17^. However, for women with a risk of FL, the migratory response of decidualized endometrial cells is not inhibited in embryos with a poor morphology. According to molecular evidence, in women with recurrent pregnancy loss (RPL), endometrial cells downregulate the expression of mucin-1, which is a regulator of embryonic implantation, and thereby prevent the attachment of embryos with a poor morphology to the endometrium^18,19^. In addition, aberrant expression of decidual markers, such as prolactin and prokineticin-1, indicates impaired decidualization, which extends the receptivity window for the implantation of low-viability embryos^10,36^. These findings suggest that selecting blastocysts with a favorable quality for ET not only influences implantation and pregnancy outcomes but also mitigates the risk of FL after implantation^34,37^. Our results indicate that the cycle and patient characteristics were similar between the D5 and D6 implanted blastocyst groups (Table 2). Under these conditions, TL analysis indicated a low frequency of embryonic dysmorphisms in implanted embryos selected by PGT-A and no substantial differences between the D5 and D6 groups, with the exception of the multinucleation at the 4-cell stage (Table 3). Moreover, a comparison of the embryonic kinetics in the groups with implanted D5 vs. D6 blastocysts revealed considerably delayed cell division during both the early cleavage and blastocyst stages, prolonged intervals of blastocyst formation, and lower scores of KIDScore™ D5 model in the D6 group (Table 4). Nevertheless, logistic regression analysis revealed that the probability of LB was not associated with any dysmorphisms or developmental kinetics (Table 6). In a previous study, a mouse model demonstrated that asynchronous cell division during the early cleavage stage affects embryonic compaction and cell lineage formation through the aberrant nuclear translocation of yes-associated protein 1, resulting in a reduced cell number with ICM and an increased risk of abortion^38^. However, a large body of evidence has indicated that the morphokinetic profiles of FL and LB embryos are indistinguishable^39^. McQueen et al. used TL imaging to compare the morphokinetics between euploid embryos resulting in clinical FL, biochemical FL, and LB in patients undergoing IVF. Similar to our findings, they reported that the embryonic morphokinetic parameters cannot predict FL in PGT-A cycles^40^.

In the present study, using uniform time point assessments along with TL monitoring, we discovered substantial differences in the blastocyst morphological components between the D5 and D6 implanted blastocyst groups. In contrast to the developmental kinetics, positive correlations between the probability of LB and ICM or TE grading were observed in successfully implanted embryos (Table 7). Shi et al. reported considerably different FL rates between blastocysts classified by TE grading (grade A vs. grade B vs. grade C, 15.3% vs. 11.8% vs. 9.8%) in young IVF patients who received embryos without PGT-A and had a positive intrauterine pregnancy^34^. Moreover, in accordance with the findings of the present study, several reports have indicated that the grade of ICM or TE morphology in euploid embryos with FL is inferior to that in embryos with LB, indicating that blastocyst morphology is a critical biomarker associated with FL in euploid transfer cycles^40,41^. Recent reports have revealed better clinical outcomes in blastocysts with equal morphological quality on D5 than on D6, regardless of the PGT-A status^22,23,29^. The refined evaluation of blastocyst morphology based on TL monitoring at a uniform time point in the present study also demonstrated that blastocysts with a favorable morphology had a higher LB rate than that of blastocysts with a poor morphology. However, the clinical outcomes were similar between D5 and D6 blastocysts with an indistinguishable quality of morphology.

The primary limitation of the present single-center study was its retrospective nature. The absence of randomization may have resulted in selection bias, and 14 of the 304 enrolled patients had undergone multiple cycles of ET. Therefore, in this study, we analyzed repeated measurements by introducing the GEE model, which can be used to address the problem of potential correlations within the same subjects and is a marginal model that is widely adopted for longitudinal data. Although no major confounding variables related to LB were detected in the data set, a multivariate logistic regression model was used to confirm the correlation between LB and the dysmorphisms, development speed, and morphology of blastocysts by adjusting the candidate variables (i.e., AMH, serum estradiol levels, oocyte sources, and ploidy status; Table 1).

Despite the promising clinical improvement offered by PGT-A, FL may still occur in patients who undergo successful IVF. Even if patients experience RPL, research has indicated that embryonic quality may play an essential role in postimplantation development, indicating the necessity of extensive exploration of the embryonic factors that can predict FL in PGT-A cycles. In conclusion, using TL monitoring, we discover that D6 blastocysts are of a lower quality, which may affect the LB rate of implanted embryos, than that of D5 blastocysts. We also demonstrate that intrinsic embryonic factors are more likely to be the morphology of ICM and TE rather than dysmorphisms or morphokinetics.

### Supplementary Information

Supplementary Information.

## Data Availability

The datasets generated and/or analysed during the current study are available in the NCBI SRA repository (PRJNA937335, https://www.ncbi.nlm.nih.gov/sra). The reviewer link: https://dataview.ncbi.nlm.nih.gov/object/PRJNA937335?reviewer=vi5umrj0t9mqb2unrdti17q7rd.

## Abbreviations

PGT-A: Preimplantation genetic testing for aneuploidy
D5: Day 5
D6: Day 6
LB: Live birth
TL: Time-lapse
SET: Single-embryo transfer
ET: Embryo transfer
ICM: Inner cell mass
TE: Trophectoderm
IVF: In vitro fertilization
FET: Frozen embryo transfer
hr-NGS: High-resolution next-generation sequencing
FL: Fetal loss
AMH: Anti-Müllerian hormone
BMI: Body mass index
E2: Estradiol
P4: Progesterone
GnRH: Gonadotrophin-releasing hormone
hCG: Human chorionic gonadotropin
GEE: Generalized estimating equation
OR: Odds ratio
CI: Confidence interval
IL: Interleukin
RPL: Recurrent pregnancy loss
vs.: Versus
