# Supplementary material for: Increased incidence of live births in implanted day 5 versus day 6 blastocysts following single embryo transfers with PGT-A
Source: Sci Rep. 2023 Aug 5;13:12725. doi: 10.1038/s41598-023-40052-5 (PMC10404267; doi:10.1038/s41598-023-40052-5)
Supplement: Supplementary file 1 — Supplementary Information. [file 41598_2023_40052_MOESM1_ESM.docx]

Supplementary Table 1. The definitions of embryonic parameters under time-lapse monitoring.

| Parameters | Definitions |
| --- | --- |
| **Dysmorphisms** | |
| Uneven cleavage | the ratio of any two blastomere diameters < 0.75. |
| Multinucleation | appearance of any types (e.g. single, complex) of multiple nuclei within individual blastomeres. |
| Non-central juxtaposition | non-central position of pronuclei at the firstly contact timing of male and female pronuclei. |
| No pronuclear contact | no pronuclear contact just before pronuclear fading. |
| Uneven PN size | uneven sizes (the difference of pronuclear sizes ≥ 45 𝜇m^2^) between male and female pronuclei just before pronuclear fading. |
| Unsynchronized PN fading | unsynchronized fading time for male and female pronuclei. |
| Twist-and-crumble division | a zygote showing a struggling division at the first cell cycle (often with formation of irregular blebbing, membrane ruffling, or pseudo-furrows) and resulting in fragmentation (< 25%). |
| Incomplete chaotic division | a zygote showing a struggling division at the first cell cycle (often with formation of irregular blebbing, membrane ruffling, or pseudo-furrows) and resulting in fragmentation (≥ 25%). |
| Direct unequal cleavage | a single blastomere dividing directly or rapidly (< 5 h) from 1 cell to 3 cells at the first, second, or third cleavage. |
| Reverse cleavage | abnormal cell refusion at the first, second, or third cleavage. |
| Delayed cleavage | a single blastomere postponing division for at least one cell cycle at the first, second, or third cleavage. |
| Vacuolization | appearance of embryo vacuoles during *in vitro* culture. |
| Premature compaction | occurrence of embryo compaction before the 9-cell satge. |
| **Morphokinetics** | |
| tPNf | time for both pronuclei fading. |
| t2 | time for fomation of 2 cells after tPNf. |
| t3 | time for fomation of 3 cells after tPNf. |
| t4 | time for fomation of 4 cells after tPNf. |
| t5 | time for fomation of 5 cells after tPNf. |
| t8 | time for fomation of 8 cells after tPNf. |
| tM | time for embryo accomplishing compaction after tPNf. |
| tSB | time for embryo starting blastocoel formation after tPNf. |
| tB | time for blastocoel cavity starting to push zona pellucida after tPNf. |
| tM-tB | the time period between tM and tB (tB-tSB). |
| tSB-tB | the time period between tSB and tB (tB-tSB). |
| ***Blastocyst morphology** | |
| Expansion level ≤ 1 | non-blastocoel formation is observed or the embryo starts to form blastocoel. |
| Expansion level 2 | the blastocoel cavity starts to push zona pellucida. |
| Expansion level 3 | the embryo starts to herniate. |
| ICM grade C or less | The ICM is indistinguishable or very few cells form a loosely packed cell mass with distinct boundaries. Various ICM sizes may observed in this group because of uneven cell sizes and poor compaction. The layer can be not homogenous with vacuoles, degenerated cells or independent cells. |
| ICM grade B | Several cells form the a less tightly packed cell mass. The layer can be less homogenous with few vacuoles or minor degenerations. |
| ICM grade A | Many cells form a tightly packed cell mass without distinct boundaries. The layer is homogenous without vacuoles and debris. |
| TE grade C or less | The TE is indistinguishable or very few and larger cells often stretches over a large area. Cell cytoplasm often appears non-homogenous and vacuoles may be present. |
| TE grade B | Several cells (often > 20) are shown. The layer is not completely organized and the shape of the cells varies within the layer. Cell cytoplasm may appear non-homogenous and cell nuclei may be difficult to distinguish. |
| TE grade A | Many flattened cells (often > 40) forms a organized layer that lines the blastocoel cavity. Cell cytoplasm is homogenous and cells nuclei are often clearly visible. |

*The blastocyst morphology was assessed at 118 hpi and the grades of ICM and TE were evaluated for the blastocysts with the expansion levels ≥ 2.
